# Identification of the Active Compound of Liu Wei Di Huang Wan for Treatment of Gestational Diabetes Mellitus via Network Pharmacology and Molecular Docking

**DOI:** 10.1155/2022/4808303

**Published:** 2022-05-28

**Authors:** Yunqi Xiong, Qiutong Li, Xiuhui Chen, Ting Zhu, Qitian Lu, Guojing Jiang

**Affiliations:** ^1^Department of Obstetrics and Gynaecology, Shuguang Hospital Affiliated to Shanghai Traditional Chinese Medical University, Shanghai 200120, China; ^2^Department of Obstetrics and Gynaecology, Nanjing Drum Tower Hospital Affiliated to Nanjing University Medical School, Nanjing 210008, China

## Abstract

Liu Wei Di Huang Wan (LWDHW) is a well-known Chinese herbal compound, which has been prescribed for the treatment of gestational diabetes mellitus (GDM). We sought to clarify the potential therapeutic effects of LWDHW against GDM. Differentially expressed genes (DEGs) in GDM were firstly identified from the Gene Expression Omnibus (GEO) database. Gene Ontology (GO) and Kyoto Encyclopedia of Genes and Genomes (KEGG) pathway enrichment analyses were performed to reveal the biological functions of the DEGs. Subsequently, the LWDHW-compound–target network was constructed based on public databases to identify the relationship between the active components in LWDHW and the corresponding targets. Furthermore, gene functional analysis and protein–protein interaction (PPI) network construction were applied to investigate the function of potential targets and to evaluate hub genes. Finally, molecular docking was used to verify the binding activities between active ingredients and hub targets. Thirteen active components and 39 corresponding therapeutic target genes were obtained via network pharmacology analysis. The enrichment analysis demonstrated that the anti-GDM effect of LWDHW included oxidoreductase activity, involvement in renal system process, and regulation of blood pressure, which may be achieved through regulation of serotonergic synapses, vascular smooth muscle contraction, and neuroactive ligand–receptor interaction pathways. Additionally, molecular docking revealed that the main active component, Mu Dan Pi, exhibited the best affinity for proteins encoded by hub genes. This study applied network pharmacology analysis and molecular docking to display the multicomponent and multitarget characteristics of LWDHW in the treatment of GDM. Our findings provide novel insights into the pathogenesis of GDM and the therapeutic mechanisms of LWDHW against GDM.

## 1. Introduction

GDM is defined as diabetes diagnosed during pregnancy, with an approximately 2–10% morbidity in pregnant women [1, 2]. With the increasing frequency of obesity and type 2 diabetes, GDM is becoming more common. According to the results of a meta-analysis, the total incidence of GDM is 14.8% in China mainland. Older women had approximately twice the rate of GDM compared to younger women [3]. It was also proved that the incidence of GDM rose with advanced maternal age in another prospective cohort study in west China [4]. After the “two-child policy” put into effect in 2015, the prevalence of GDM has continued to increase during the past years and is likely to reach another peak in the future [5]. GDM is not only associated with adverse pregnancy outcomes, such as macrosomia and stillbirth [6], but it also increases the risk of metabolic syndrome in pregnant women and their offspring [7, 8]. Even though the multidisciplinary treatment of GDM includes modifications of lifestyle and pharmacological treatment, the most appropriate therapy for pregnancies complicated by GDM remains controversial, due to the unresolved safety issues of antidiabetic agents and uncertain treatment options [9, 10]. Therefore, the identification of effective molecular targets and elucidation of potential mechanisms related to GDM are of great importance for the improvement of the maternal and neonatal outcomes.

Traditional Chinese medicine (TCM) has been used for centuries to prevent and treat disease in China. The active components of several types of TCMs show great potential as effective therapy for GDM through different mechanisms, including the inhibitory effect on inflammation, enhancement of *β* cell function, and regulation of hepatic gluconeogenesis [11, 12].

Liu Wei Di Huang Wan (LWDHW) is a popular Chinese herbal formula and is commonly prescribed for the treatment of diabetes [13], hypertension [14], menopause [15], and systemic lupus erythematosus [16]. It is a formulation composed of six ingredients: Shu Di Huang (Radix Rehmanniae Preparata), Shan Zhu Yu (Fructus Corni), Mu Dan Pi (Cortex Moutan), Shan Yao (Rhizoma Dioscoreae), Fu Ling (Poria), and Ze Xie (Rhizoma Alismatis). However, the underlying mechanism of LWDHW in the treatment of GDM has not been fully clarified.

Network pharmacology, a methodology developed in recent years, can reveal the complex relational network of medicine, genes, and targets of diseases [17] and is able to predict and elucidate the potential mechanism of multiple components, targets, and pathways of TCMs, through the construction of the various complex networks and the analysis of multilevel connections [18]. Network pharmacology could be of great help in the development of cost-effective drug development [19] and in the identification of synergistic TCMs [20]. This would provide a novel approach to elucidating the molecular mechanisms by which LWDHW has therapeutic effects on GDM.

Here, we sought to clarify the mechanism by which LWDHW exerted an affect against GDM. We first identified genes that are differentially expressed in GDM from a Gene Expression Omnibus (GEO) database (http://www.ncbi.nlm.nih.gov/) [21]. Then, the active components of LWDHW and their corresponding target genes were predicted according to the Traditional Chinese Medicine Systems Pharmacology (TCMSP) and Bioinformatics Analysis Tool for Molecular mechanism of Traditional Chinese Medicine (BATMAN-TCM) [22, 23]. The intersection of DEGs and drug-target genes was determined. We performed enrichment analysis and established a protein–protein interaction (PPI) network to identify the hub target genes and their corresponding pathways. A LWDHW-main active compound–GDM-target–signaling pathway network was constructed. Finally, the anti-GDM effect of LWDHW, as suggested by the network analysis, was further verified by molecular docking.

## 2. Materials and Methods

### 2.1. Identification of Targets of GDM

The GDM gene expression dataset was downloaded from the GEO database (http://www.ncbi.nlm.nih.gov/) [21]. We systematically searched for microarray studies by using the terms “maternal diabetes” and “*Homo sapiens*.” The GSE51546 dataset (GPL 10558, Illumina Human HT-12 V4.0 expression beadchip) was obtained, which included data from six women with GDM and six healthy pregnant controls. Then, we downloaded the platform annotation file and series matrix files. The data were normalized, and each corresponding gene probe was converted into a gene symbol by using the Bioconductor R package [24]. If there were multiple probes that could be mapped to the same gene symbol, the average was applied. Genes that were differentially expressed (DEGs) between the maternal diabetes tissues and normal control tissues in the microarray were identified by using the “limma” (linear models for microarray data) program in the R package [25]. In order to correct the *P* value and control the false discovery rate, the Benjamini–Hochberg method was applied for multiple comparisons. A |log_2_ fold change (FC)| >0.263 and a *P* value < 0.05 were regarded as the cut-off criteria for DEGs. Heatmap and volcano plots of significant DEGs were generated using the R software.

### 2.2. Functional and Pathway Enrichment Analyses

Gene Ontology (GO) displays gene functions from three different aspects: molecular function (MF), cellular component (CC), and biological process (BP) [26]. Kyoto Encyclopedia of Genes and Genomes (KEGG) [27] is a practical database established in 1995 by Kyoto University Kanehisa Laboratory of Bioinformatics Center. GO and KEGG pathway enrichment analyses were performed for the DEGs using the clusterProfiler R package [28]. A *P* value < 0.05 was considered to be statistically significant. The results of GO and KEGG enrichment analyses were displayed as bubble charts generated by the ggplot_2_ R package.

### 2.3. Identification of the LWDHW Active Ingredients

The constituents of LWDHW were entered into the TCMSP database (https://tcmspw.com/tcmsp.php) [22] to obtain properties including the molecular name, molecular weight, lipid–water partition coefficient, number of donor atoms of H-bonds and acceptor atoms for H-bonds, oral bioavailability (OB), ability to cross the blood–brain barrier drug-likeness (DL), and drug half-life (HF). In order to obtain the absorption, distribution, metabolism, and excretion (ADME) properties of compounds [29, 30], OB, DL, and HF were calculated to screen the potential active components. OB ≥ 20% and DL ≥ 0.1 were used as the criteria for choosing candidate compounds for further analysis.

### 2.4. Construction of PPI Network

The STRING database (http://string-db.org/) was used to establish a PPI network [31]. In the PPI analysis, a combined score > 0.15 was regarded as a significant interaction criterion. The PPI network was visualized with Cytoscape 3.8.0 (https://cytoscape.org/) [32]. Additionally, the novel Cytoscape plugin CytoHubba [33] was used to screen hub genes in the PPI network.

### 2.5. Active Compound of LWDHW and Corresponding Predicted Target Genes

The corresponding protein targets of each active ingredient in LWDHW were predicted using the BATMAN-TCM (http://bionet.ncpsb.org/batman-tcm/) database, which is the first online bioinformatics analysis tool specifically designed for research into the molecular mechanisms of TCMs [23]. Then, the LWDHW-compound and LWDHW-compound–target networks were constructed and visualized using Cytoscape software.

### 2.6. Functional Enrichment Analysis of LWDHW-Related Targets

The intersection of DEGs in GDM and the corresponding targets of the active compounds were obtained by using a Venn diagram. Then, to investigate the biological function of the potential targets, we performed GO and KEGG pathway enrichment analyses using the clusterProfiler R package and visualized the results by means of the ggplot_2_ R package, as before.

### 2.7. Construction of the LWDHW-Main Active Compound–GDM-Target-Signaling Pathway Network

To identify the potential GDM-related targets for treatment, a Venn diagram was applied to define the intersection of LWDHW targets and GDM-related targets. The corresponding chemical compounds of the intersecting targets were considered possible therapeutic ingredients for the treatment of GDM. Then, the LWDHW-main active compound–GDM-target-signaling pathway network was constructed to identify the potential relationship between the active compounds of LWDHW and their corresponding targets, which was then visualized as described above.

### 2.8. Verification through Molecular Docking

To further elucidate the underlying mechanism of LWDHW in the treatment of GDM, molecular docking was applied to predict and verify the binding activity of the core active compounds of LWDHW to proteins encoded by the hub genes with the top three Matthews correlation coefficient (MCC) scores. These genes were considered potential GDM-related targets. First, the SDF format file of the active component was obtained from the PubChem database (https://pubchem.ncbi.nlm.nih.gov/) and converted into mol_2_ format by PyMoL. Then, the PDB format file of the proteins encoded by the hub genes were downloaded from PDB database. Finally, Autodock Vina software [34], which improves the average accuracy of combined model prediction and performs ligand–protein molecular docking, was used to analyze the binding affinities and predict virtual docking.

### 2.9. Statistical Analysis

All statistical analysis were performed with R (version 3.6.3; https://www.r-project.org/) and were presented as mean ± standard deviation. All statistical tests were two-tailed. Differences were considered significant at *P* < 0.05.

## 3. Results

### 3.1. Target Prediction of GDM

**Figure 1:**
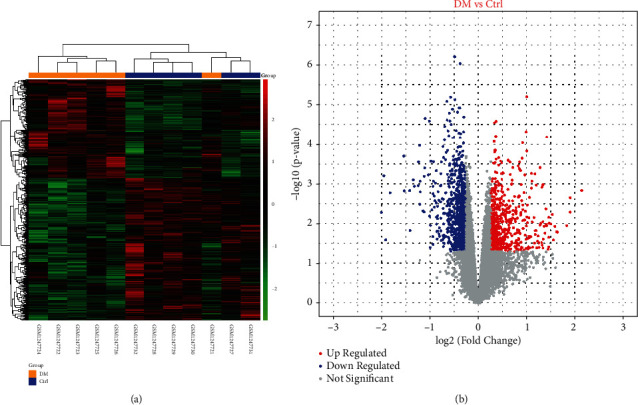
(a) Heatmap of differentially expressed genes (DEGs) from the microarray analysis. The orange group is the gestational diabetes mellitus (GDM) group, while the blue group is the control group. The upregulated genes are shown in red, and the downregulated genes are shown in blue. (b) DEGs obtained from the microarray. The red points represent upregulated genes, and blue points represent downregulated genes. Gray points represent genes without a significant difference in expression.

We identified 1408 genes with differential expression in GDM from GSE51546. As shown in Figure 1, there were 574 upregulated genes and 834 downregulated genes. These DEGs were considered the potential GDM therapeutic targets and were further analyzed.

### 3.2. Enrichment Analysis of DEGs in GDM

**Figure 2:**
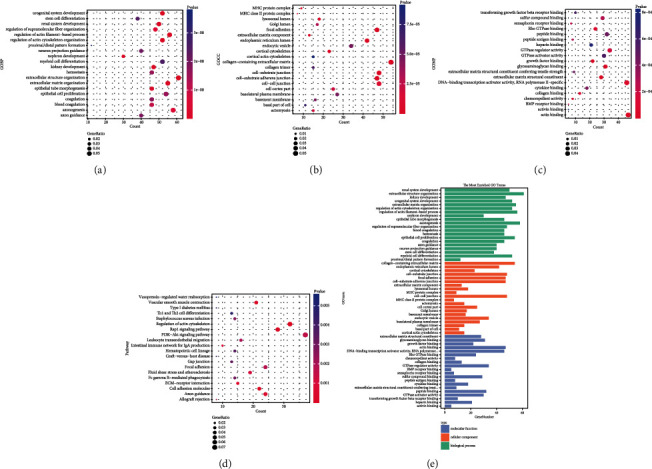
Enrichment analysis of the differentially expressed genes of gestational diabetes mellitus (GDM). (a) Bubble plot of the top 20 enriched biological process (BP). (b) Cellular component (CC) and (c) molecular function (MF) terms revealed by Gene Ontology (GO) functional enrichment analysis of differentially expressed genes (DEGs) in GDM. (d) Kyoto Encyclopedia of Genes and Genomes (KEGG) pathway analysis. (e) Bar plot of the top 20 enriched GO terms. The size of the bubble indicates the gene count, while colors indicate the significance of enrichment.

The top 20 enriched GO terms are displayed as bubble plots (Figures 2(a)–2(c)) and a bar plot (Figure 2(e)). In terms of BP, DEGs were mainly enriched for renal system development, extracellular structure organization, and kidney development. In terms of CC, DEGs were mainly enriched for the collagen-containing extracellular matrix, endoplasmic reticulum lumen, and cortical cytoskeleton. For the MF category, the enriched terms included extracellular matrix structural constituent, glycosaminoglycan binding, and growth factor binding. Furthermore, as shown in Figure 2(d), KEGG pathway enrichment analysis demonstrated the top 20 enriched pathways of the DEGs. These mainly involved the regulation of the actin cytoskeleton, RAP1 signaling pathway, and vascular smooth muscle contraction.

### 3.3. Identification of Active Compounds of LWDHW and Construction of Networks

**Figure 3:**
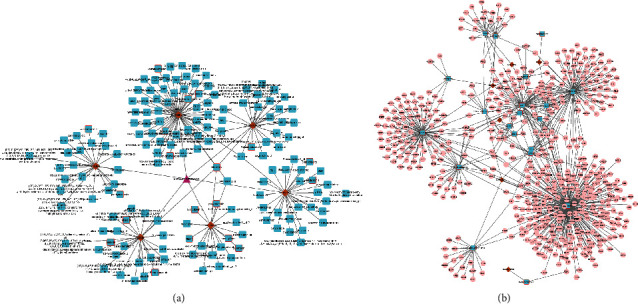
(a) LWDHW-compound network. (b) LWDHW-compound–target network. The purple triangle represents the LWDHW formulation. The brown diamonds represent the herbs, and the blue squares indicate the constituent compounds. The blue squares with the red frames indicate the main ingredients. Pink circles represent the targets.

From the TCMSP database, there were six types of herbs and 508 related components of LWDHW in total. According to the ADME thresholds, 169 active ingredients were screened (Supplementary Table S1). The LWDHW-compound network was constructed using Cytoscape 3.8.0 (Figure 3(a)). The predicted active ingredients with potential targets were selected from the BATMAN database. Finally, 19 active ingredients (Table 1) and 536 related targets of the active ingredients were obtained (Supplementary Table S2). The LWDHW-compound–target network was established and is displayed in Figure 3(b).

### 3.4. Prediction and Enrichment Analyses of the Targets

**Figure 4:**
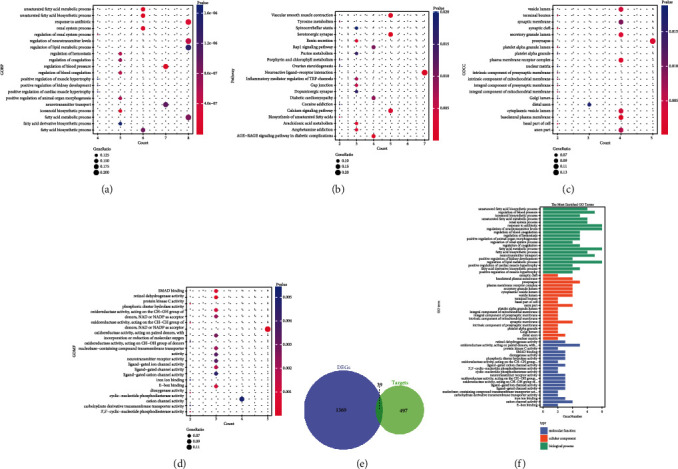
Enrichment analysis of gestational diabetes mellitus- (GDM-) related target genes. (a) Bubble plot of the top 20 enriched biological process (BP), (b) cellular component (CC), and (c) molecular function (MF) categories. (d) Kyoto Encyclopedia of Genes and Genomes (KEGG) pathway analysis. (e) Venn diagram of the intersection of differentially expressed genes (DEGs) of GDM and LWDHW targets. (f) Bar plot of the top 20 enriched Gene Ontology (GO) terms. The size of the bubble indicates the gene count, while colors indicate enrichment significance.

For further identification of the potential targets to improve treatment of GDM, the intersection of the DEGs of GDM and the active components of the LWDHW corresponding targets is shown in Figure 4. Thirty-nine genes were selected, and enrichment analysis of these genes was performed. The top 20 enriched GO terms were displayed as the bubble plots (Figures 4(a)–4(c)) and bar plot (Figure 4(e)). In terms of BP, genes were mainly enriched in renal system processes, response to antibiotics, and regulation of blood pressure. For the CC category, the enriched terms were basolateral plasma membrane, presynapse, and plasma membrane receptor complex. For the MF category, the enriched terms included oxidoreductase activity, retinol dehydrogenase activity, and SMAD binding.

**Figure 5:**
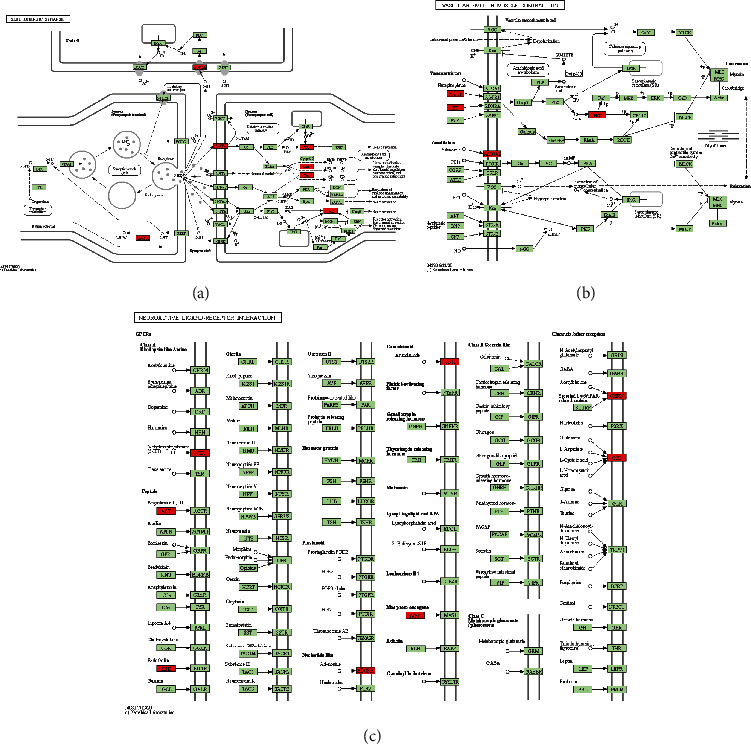
(a) hsa04726: serotonergic synapse pathway. (b) hsa04270: vascular smooth muscle contraction pathway. (c) hsa04080: neuroactive ligand–receptor interaction pathway. Red represents the critical genes involved in the pathways.

The bubble plot of the top 20 enriched pathways is shown in Figure 4(d). The top three enriched pathways of the targets were the serotonergic synapse pathway, vascular smooth muscle contraction pathway, and neuroactive ligand–receptor interaction pathway. The detailed information of the pathways and related critical genes are shown in Figure 5.

### 3.5. PPI Network Visualization

**Figure 6:**
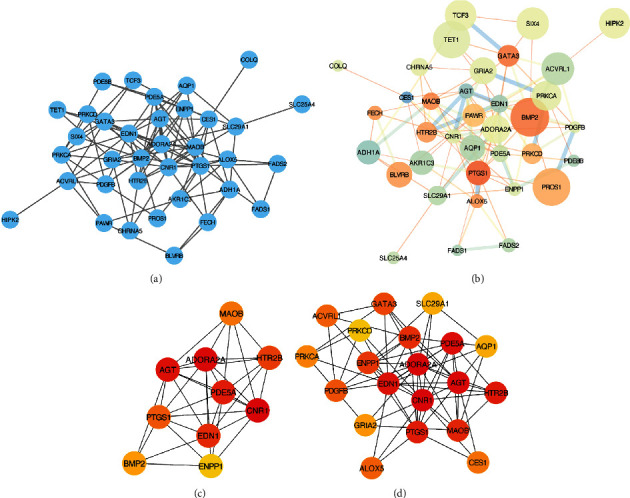
Protein–protein interaction (PPI) network of gestational diabetes mellitus (GDM) target genes. (a) PPI network, (b) network analyzer network, (c) top 10 hub genes, and (d) top 20 hub genes. The size of the circle represents the significance. From blue to red represents the different log_2_ fold change, the thickness of the connection represents the combined score.

The PPI network was constructed and visualized by using the Cytoscape software (version 3.8.0). As shown in Figures 6(a) and 6(b), the resulting network included 36 nodes and 110 edges. To identify hub target genes that may play key roles during the progression of GDM, CytoHubba was applied to rank the nodes in the PPI network according to the topological analysis method with MCC (Table 2). The top 10 hub genes and regulated genes are shown in Figure 6(c), while the top 20 hub genes and regulated genes are shown in Figure 6(d).

### 3.6. Construction of LWDHW-Main Active Compound–GDM-Target-Signaling Pathway Network

**Figure 7:**
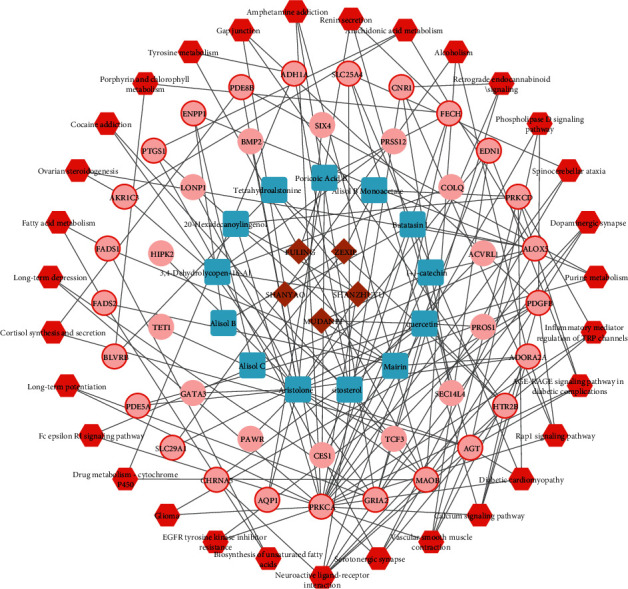
LWDHW-main active compound–GDM-target-signaling pathway network. Brown diamonds represent herbs, blue squares represent ingredients, pink circles represent target genes, and red hexagons represent pathways. The pink circles with red frames represent the pathway-related targets.

The LWDHW-main active compound–GDM-target-signaling pathway network was established for better elucidation of the therapeutic effect. As shown in Figure 7, the network contained five herbs: Mu Dan Pi, Shan Yao, Ze Xie, Shan Zhu Yu, and Fu Ling, 13 different ingredients, 39 target genes, and 31 corresponding pathways.

### 3.7. Verification by Molecular Docking

**Figure 8:**
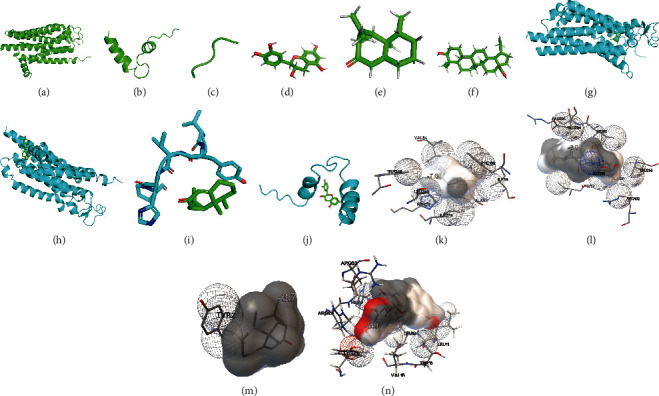
Molecular docking models of LWDHW binding to the proteins encoded by the top three hub target genes. (a) ADORA2A, (b) CNR1, (c) AGT, (d) catechin, (e) aristolone, (f) mairin, (g) ADORA2A and aristolone, (h) ADORA2A and mairin, (i) AGT and aristolone, (j) CNR1 and catechin, (k) ADORA2A and aristolone, (l) ADORA2A and mairin, (m) AGT and aristolone, and (n) CNR1 and catechin. Hydrogen bonded atoms in the receptor or atoms in close contact with atoms in the ligand are shown as spheres, and fragments of the secondary structure are shown as sequences of three or more residues interacting with the ligand in the receptor.

Molecular dynamics simulation offers novel insights into the stability and binding activities of protein–ligand complexes. Thus, to validate the potential GDM therapeutic targets, we performed molecular docking of LWDHW with the proteins encoded by the top three hub genes. Docking analysis predicted Vina scores successfully. Specific information is shown in Table 3. Furthermore, structure matching analysis was performed by using PyMoL software. Finally, we obtained four groups: AGT and aristolone, ADORA2A and mairin, ADORA2A and aristolone, and CNR1 and catechin, as shown in Figure 8. A Vina score < −5 was considered to be convincing. In particular, molecular docking between ADORA2A and mairin had the lowest Vina score: -8.6. ADORA2A was the target of Mu Dan Pi and Shan Zhu Yu. Mairin is an important ingredient of Mu Dan Pi. Overall, the results of molecular docking analysis indicated that the active components of LWDHW had good binding activities to the proteins encoded by the top three hub genes.

## 4. Discussion

In this study, we sought to clarify the potential therapeutic effects of LWDHW against GDM, by investigating the intersection of GDM DEGs and LWDHW targets, followed by virtual docking experiments for verification. Molecular docking analysis indicated that the active components of LWDHW bound well to the proteins encoded by the top three hub genes. Specifically, aristolone (an active compound of Shan Zhu Yu) targeted AGT and ADORA2A, mairin (a main ingredient of Mu Dan Pi) also targeted ADORA2A, while catechin targeted CNR1.

GDM is the commonest medical complication during pregnancy and imposes a marked economic and health burden with the increasing prevalence of obesity [5]. Since oral antidiabetic agents have side effects and safety issues, the management and treatment of GDM still remain challenging. Thus, a novel therapeutic strategy for GDM is urgently needed [35]. With a long history of use and effectiveness, TCM has increasingly attracted global attention for its unique insights into pathogenesis and multitarget treatment. Growing evidence has shown the potential function of LWDHW in the treatment of diabetes. A population-based case–control study of patients with type 2 diabetes has indicated that LWDHW can relieve diabetic nephropathy [36]. Another study in a mouse model showed its beneficial effects in diabetes-related renal failure [37]. The use of LWDHW and oral antidiabetic drugs have been reported to be associated with delayed use of insulin [38]. To elucidate the potential function and evaluate the beneficial effects of LWDHW on GDM further, the present study investigated the active ingredients and potential mechanisms of this TCM comprehensively using network pharmacology.

We identified 19 active compounds in LWDHW, 508 drug-related targets, and 1408 DEGs of GDM from public databases. Among these, there were 39 genes in common between the DEGs and the drug-corresponding targets, suggesting their potential role in anti-GDM action. In the PPI network of these 39 target genes, the proteins were not independent but were linked in a network. After evaluation of the compound and construction of the network, 13 active compounds, 39 targets, and 31 pathways were finally identified (Figure 7). These results suggested that LWDHW has multitarget biological function, with its multiple compounds, in the treatment of GDM.

LWDHW has displayed beneficial effects on alleviation of insulin resistance through regulating the PI3K/AKT signaling pathway, according to previous studies [39, 40]. In contrast, in our research, the GO and KEGG enrichment analyses suggested that the top three signaling pathways were the serotonergic synapse pathway, vascular smooth muscle contraction pathway, and neuroactive ligand–receptor interaction pathway, which are involved in the progression of GDM and could underlie the mechanism by which LWDHW exerts a therapeutic effect on GDM. It has been acknowledged that 5-hydroxytryptamine (5-HT) might play a significant role in the etiology of GDM [41], which could be a promising biomarker and potential risk factor of GDM [42]. A recent study explained that 5-HT uptake rates is decreased in GDM trophoblasts, resulting in defective insulin signaling and glycosylation [43]. Additionally, it has been reported that arterial dilation in obese women with GDM is attenuated, which is likely due to both endothelial and smooth muscle dysfunction [44]. Thus, the regulation of 5-HT and vascular smooth muscle contraction may be promising therapeutic strategies for GDM. According to a recent network pharmacology analysis [45], LWDHW shows better effects on the function of neuroendocrine immunity, from a holistic point of view, than donepezil, memantine, and melatonin. Nevertheless, the correlation between the neuroactive ligand–receptor interaction pathway and GDM requires further investigation.

Additionally, molecular docking further verified the possible combinations of compounds and target hub genes (Figure 8). According to our analysis, mairin, which is the main component of Mu Dan Pi, was the most active compound with the highest binding affinity, for the target ADORA2A. Ample evidence has proven that Mu Dan Pi has a wide range of functions, such as antioxidant, antidiabetic, antitumor, and antioxidant effects [46]. In a study of type 2 diabetes mellitus, the active compounds from Mu Dan Pi were shown to activate the AMPK pathway and reverse metabolic abnormalities [47]. Consistently, we also found that mairin in Mu Dan Pi may be a promising component due to its affinity for GDM-related targets. ADORA2A encodes the adenosine receptor A2a. ADORA2A-mediated signaling results in the breakdown of the blood–brain barrier, which is induced by obesity. The mechanism implicates cerebrovascular dysfunction in diabetes [48]. A recent study has indicated that adenosine signaling via ADORA2A is increased in compensation of *β* cell proliferation, which could strengthen the expansion of *β* cells pharmacologically [49]. Taken together, the ADORA2A/mairin combination could be a promising therapeutic strategy for diabetes in future.

Our study has yielded marked insights into the molecular mechanisms by which LWDHW exerts therapeutic effects in GDM. Nevertheless, the current study had some limitations. First, the one-sided results of single microarray analysis may lead to a high false positive rate; thus, it is necessary to improve the power of detection by integrating the results of analysis of multiple datasets. Second, although bioinformatics analysis is a powerful tool for the identification of potential targets in GDM and to improve mechanistic understanding, the therapeutic effect and potential mechanisms of active components still need to be validated in further experimental research. Third, to clarify whether LWDHW can be beneficial to maternal and neonatal outcomes, the safety of this drug should be evaluated.

## 5. Conclusions

In summary, our research elucidated the potential active ingredients and multiple target mechanisms of LWDHW in the treatment of GDM, which have not been published previously and which provide scientific evidence for clinical practice and application of LWDHW for further research. Our results clarified the potential therapeutic mechanism of the compound and verified this with molecular docking. The key targets, pathways, and ingredients identified here form a basis for further research and facilitate herbal medicine use for the treatment of GDM in future. However, due to the limitations of network pharmacology analysis, it needs to be established whether the active components themselves could be used in GDM treatment in future. Additionally, more clinical research and molecular experiments are necessary to validate our current findings.

## Figures and Tables

**Table 1:** The active compounds of Liu Wei Di Huang Wan.

Molecule name	MW	OB	DL
Alisol B	472.78	34.47	0.82
Alisol C	486.76	32.7	0.82
Alisol B monoacetate	514.82	35.58	0.81
Rehmannioside A	524.53	25.95	0.87
Pachymic acid	528.85	33.63	0.81
Poricoic acid B	484.74	30.52	0.75
Batatasin I	284.33	23.7	0.27
Campesterol	400.76	37.58	0.71
Mairin	456.78	55.38	0.78
Quercetin	302.25	46.43	0.28
Sitosterol	414.79	36.91	0.75
Paeonidanin	492.57	24.64	0.78
Kaempferol	286.25	41.88	0.24
(+)-Catechin	290.29	54.83	0.24
Tetrahydroalstonine	352.47	32.42	0.81
20-Hexadecanoylingenol	586.94	28.2	0.68
3,4-Dehydrolycopen-16-al	548.92	46.64	0.49
Aristolone	218.37	45.31	0.13
Cornudentanone	378.56	39.66	0.33

Abbreviations: MW: molecular weight; OB: oral bioavailability; DL: drug likeness.

**Table 2:** Top 10 hub genes according to the Matthews correlation coefficient (MCC).

Gene	MCC score	Degree	Closeness	Betweenness
CNR1	416	15	24.5	192.09991
ADORA2A	404	12	22.83333	76.40726
AGT	392	11	22.33333	53.54971
PDE5A	386	9	20.83333	28.23189
EDN1	296	15	24.33333	169.122
HTR2B	242	7	19.16667	11.27673
PTGS1	176	13	23.5	176.15779
MAOB	132	9	20.25	44.90227
BMP2	38	10	21.33333	81.15432
ENPP1	34	7	20	33.68954

**Table 3:** List of the best models for molecular docking.

Herb name	Active ingredient	Target gene	Affinity
Mu Danpi	Mairin	ADORA2A	-8.6
Mu Danpi	(+)-Catechin	CNR1	-5.9
Shan Zhuyu	Aristolone	ADORA2A	-7.6
Shan Zhuyu	Aristolone	AGT	-3.7

## Data Availability

The dataset (GSE51546) applied for this study can be found in the Gene Expression Omnibus (GEO) database (http://www.ncbi.nlm.nih.gov/). The data involved in this study are available from the corresponding author by request.

## Supplementary Materials

Supplementary Materials Supplementary Table S1: active ingredients of LWDHW screened out from TCM SP. Supplementary Table S2: active ingredients and corresponding targets.
