# Supplementary material for: Identification of the Active Compound of Liu Wei Di Huang Wan for Treatment of Gestational Diabetes Mellitus via Network Pharmacology and Molecular Docking
Source: J Diabetes Res. 2022 May 28;2022:4808303. doi: 10.1155/2022/4808303 (PMC9167086; doi:10.1155/2022/4808303)
Supplement: Supplementary Materials — Supplementary Table S1: active ingredients of LWDHW screened out from TCM SP. Supplementary Table S2: active ingredients and corresponding targets. [file 4808303.f1.docx]

| **Supplementary table S2. Active ingredients and corresponding targets** | | | | |
| --- | --- | --- | --- | --- |
| herb | molecule | Gene ID | Symbol | Score |
| ZEXIE | Alisol B | 5734 | PTGER4 | 48 |
| ZEXIE | Alisol B | 5732 | PTGER2 | 48 |
| ZEXIE | Alisol B | 5733 | PTGER3 | 48 |
| ZEXIE | Alisol B | 10381 | TUBB3 | 23 |
| ZEXIE | Alisol B | 22978 | NT5C2 | 23 |
| ZEXIE | Alisol B | 132 | ADK | 23 |
| ZEXIE | Alisol B | 3614 | IMPDH1 | 23 |
| ZEXIE | Alisol B | 5167 | ENPP1 | 23 |
| ZEXIE | Alisol C | 5734 | PTGER4 | 48 |
| ZEXIE | Alisol C | 367 | AR | 48 |
| ZEXIE | Alisol C | 5732 | PTGER2 | 48 |
| ZEXIE | Alisol C | 5733 | PTGER3 | 48 |
| ZEXIE | Alisol C | 4306 | NR3C2 | 48 |
| ZEXIE | Alisol C | 10381 | TUBB3 | 23 |
| ZEXIE | Alisol C | 22978 | NT5C2 | 23 |
| ZEXIE | Alisol C | 132 | ADK | 23 |
| ZEXIE | Alisol C | 3614 | IMPDH1 | 23 |
| ZEXIE | Alisol C | 5167 | ENPP1 | 23 |
| ZEXIE | Alisol B Monoacetate | 5578 | PRKCA | 48 |
| ZEXIE | Alisol B Monoacetate | 3156 | HMGCR | 48 |
| ZEXIE | Alisol B Monoacetate | 3689 | ITGB2 | 48 |
| ZEXIE | Alisol B Monoacetate | 3683 | ITGAL | 48 |
| ZEXIE | Alisol B Monoacetate | 3066 | HDAC2 | 48 |
| ZEXIE | Alisol B Monoacetate | 5580 | PRKCD | 48 |
| SHUDIHUANG | Rehmannioside A | 5422 | POLA1 | 23 |
| FULING | Pachymic Acid | 5241 | PGR | 122.778 |
| FULING | Pachymic Acid | 367 | AR | 122.778 |
| FULING | Pachymic Acid | 4306 | NR3C2 | 122.778 |
| FULING | Pachymic Acid | 3156 | HMGCR | 80.882 |
| FULING | Pachymic Acid | 2099 | ESR1 | 48 |
| FULING | Pachymic Acid | 5734 | PTGER4 | 48 |
| FULING | Pachymic Acid | 2908 | NR3C1 | 48 |
| FULING | Pachymic Acid | 3689 | ITGB2 | 48 |
| FULING | Pachymic Acid | 5732 | PTGER2 | 48 |
| FULING | Pachymic Acid | 5733 | PTGER3 | 48 |
| FULING | Pachymic Acid | 5737 | PTGFR | 48 |
| FULING | Pachymic Acid | 301 | ANXA1 | 48 |
| FULING | Pachymic Acid | 3683 | ITGAL | 48 |
| FULING | Pachymic Acid | 1588 | CYP19A1 | 48 |
| FULING | Pachymic Acid | 3066 | HDAC2 | 48 |
| FULING | Pachymic Acid | 4058 | LTK | 22.373 |
| FULING | Pachymic Acid | 7157 | TP53 | 22.373 |
| FULING | Pachymic Acid | 815 | CAMK2A | 22.373 |
| FULING | Pachymic Acid | 1401 | CRP | 22.373 |
| FULING | Pachymic Acid | 3630 | INS | 22.373 |
| FULING | Pachymic Acid | 57103 | TIGAR | 22.373 |
| FULING | Pachymic Acid | 817 | CAMK2D | 22.373 |
| FULING | Pachymic Acid | 134 | ADORA1 | 22.373 |
| FULING | Poricoic Acid B | 2099 | ESR1 | 48 |
| FULING | Poricoic Acid B | 5241 | PGR | 48 |
| FULING | Poricoic Acid B | 367 | AR | 48 |
| FULING | Poricoic Acid B | 2908 | NR3C1 | 48 |
| FULING | Poricoic Acid B | 301 | ANXA1 | 48 |
| FULING | Poricoic Acid B | 2104 | ESRRG | 23 |
| FULING | Poricoic Acid B | 1345 | COX6C | 23 |
| FULING | Poricoic Acid B | 1329 | COX5B | 23 |
| FULING | Poricoic Acid B | 1350 | COX7C | 23 |
| FULING | Poricoic Acid B | 4512 | COX1 | 23 |
| FULING | Poricoic Acid B | 1646 | AKR1C2 | 23 |
| FULING | Poricoic Acid B | 125 | ADH1B | 23 |
| FULING | Poricoic Acid B | 9377 | COX5A | 23 |
| FULING | Poricoic Acid B | 4514 | COX3 | 23 |
| FULING | Poricoic Acid B | 1346 | COX7A1 | 23 |
| FULING | Poricoic Acid B | 2235 | FECH | 23 |
| FULING | Poricoic Acid B | 1327 | COX4I1 | 23 |
| FULING | Poricoic Acid B | 5319 | PLA2G1B | 23 |
| FULING | Poricoic Acid B | 1645 | AKR1C1 | 23 |
| FULING | Poricoic Acid B | 1339 | COX6A2 | 23 |
| FULING | Poricoic Acid B | 3156 | HMGCR | 23 |
| FULING | Poricoic Acid B | 126 | ADH1C | 23 |
| FULING | Poricoic Acid B | 1340 | COX6B1 | 23 |
| FULING | Poricoic Acid B | 2172 | FABP6 | 23 |
| FULING | Poricoic Acid B | 1066 | CES1 | 23 |
| FULING | Poricoic Acid B | 1349 | COX7B | 23 |
| FULING | Poricoic Acid B | 4513 | COX2 | 23 |
| FULING | Poricoic Acid B | 1351 | COX8A | 23 |
| FULING | Poricoic Acid B | 9971 | NR1H4 | 23 |
| SHANYAO | Batatasin I | 7299 | TYR | 80.882 |
| SHANYAO | Batatasin I | 1638 | DCT | 55.444 |
| SHANYAO | Batatasin I | 7306 | TYRP1 | 55.444 |
| SHANYAO | Batatasin I | 266629 | SEC14L3 | 23 |
| SHANYAO | Batatasin I | 5515 | PPP2CA | 23 |
| SHANYAO | Batatasin I | 5578 | PRKCA | 23 |
| SHANYAO | Batatasin I | 8856 | NR1I2 | 23 |
| SHANYAO | Batatasin I | 240 | ALOX5 | 23 |
| SHANYAO | Batatasin I | 5516 | PPP2CB | 23 |
| SHANYAO | Batatasin I | 23541 | SEC14L2 | 23 |
| SHANYAO | Batatasin I | 1606 | DGKA | 23 |
| SHANYAO | Batatasin I | 5579 | PRKCB | 23 |
| SHANYAO | Batatasin I | 284904 | SEC14L4 | 23 |
| SHANYAO | Batatasin I | 8943 | AP3D1 | 22.373 |
| SHANYAO | Batatasin I | 4948 | OCA2 | 22.373 |
| SHANYAO | Batatasin I | 4935 | GPR143 | 22.373 |
| SHANYAO | Campesterol | 2099 | ESR1 | 122.778 |
| SHANYAO | Campesterol | 5241 | PGR | 122.778 |
| SHANYAO | Campesterol | 367 | AR | 48 |
| SHANYAO | Campesterol | 2908 | NR3C1 | 48 |
| SHANYAO | Campesterol | 301 | ANXA1 | 48 |
| SHANYAO | Campesterol | 7421 | VDR | 48 |
| MUDANPI | Mairin | 148 | ADRA1A | 124 |
| MUDANPI | Mairin | 154 | ADRB2 | 124 |
| MUDANPI | Mairin | 150 | ADRA2A | 124 |
| MUDANPI | Mairin | 146 | ADRA1D | 124 |
| MUDANPI | Mairin | 147 | ADRA1B | 124 |
| MUDANPI | Mairin | 152 | ADRA2C | 124 |
| MUDANPI | Mairin | 1621 | DBH | 124 |
| MUDANPI | Mairin | 217 | ALDH2 | 124 |
| MUDANPI | Mairin | 805 | CALM2 | 124 |
| MUDANPI | Mairin | 808 | CALM3 | 124 |
| MUDANPI | Mairin | 151 | ADRA2B | 124 |
| MUDANPI | Mairin | 801 | CALM1 | 124 |
| MUDANPI | Mairin | 18 | ABAT | 48 |
| MUDANPI | Mairin | 2104 | ESRRG | 48 |
| MUDANPI | Mairin | 1345 | COX6C | 48 |
| MUDANPI | Mairin | 1329 | COX5B | 48 |
| MUDANPI | Mairin | 6546 | SLC8A1 | 48 |
| MUDANPI | Mairin | 1350 | COX7C | 48 |
| MUDANPI | Mairin | 4512 | COX1 | 48 |
| MUDANPI | Mairin | 2161 | F12 | 48 |
| MUDANPI | Mairin | 1646 | AKR1C2 | 48 |
| MUDANPI | Mairin | 7442 | TRPV1 | 48 |
| MUDANPI | Mairin | 6718 | AKR1D1 | 48 |
| MUDANPI | Mairin | 9377 | COX5A | 48 |
| MUDANPI | Mairin | 4514 | COX3 | 48 |
| MUDANPI | Mairin | 367 | AR | 48 |
| MUDANPI | Mairin | 1346 | COX7A1 | 48 |
| MUDANPI | Mairin | 2235 | FECH | 48 |
| MUDANPI | Mairin | 1327 | COX4I1 | 48 |
| MUDANPI | Mairin | 5319 | PLA2G1B | 48 |
| MUDANPI | Mairin | 1339 | COX6A2 | 48 |
| MUDANPI | Mairin | 9415 | FADS2 | 48 |
| MUDANPI | Mairin | 7299 | TYR | 48 |
| MUDANPI | Mairin | 3992 | FADS1 | 48 |
| MUDANPI | Mairin | 126 | ADH1C | 48 |
| MUDANPI | Mairin | 5742 | PTGS1 | 48 |
| MUDANPI | Mairin | 1340 | COX6B1 | 48 |
| MUDANPI | Mairin | 2172 | FABP6 | 48 |
| MUDANPI | Mairin | 1066 | CES1 | 48 |
| MUDANPI | Mairin | 5743 | PTGS2 | 48 |
| MUDANPI | Mairin | 6785 | ELOVL4 | 48 |
| MUDANPI | Mairin | 6716 | SRD5A2 | 48 |
| MUDANPI | Mairin | 1349 | COX7B | 48 |
| MUDANPI | Mairin | 4513 | COX2 | 48 |
| MUDANPI | Mairin | 2550 | GABBR1 | 48 |
| MUDANPI | Mairin | 1351 | COX8A | 48 |
| MUDANPI | Mairin | 9971 | NR1H4 | 48 |
| MUDANPI | Mairin | 3356 | HTR2A | 26.373 |
| MUDANPI | Mairin | 135 | ADORA2A | 26.373 |
| MUDANPI | Mairin | 2099 | ESR1 | 26.373 |
| MUDANPI | Mairin | 2908 | NR3C1 | 26.373 |
| MUDANPI | Mairin | 1813 | DRD2 | 26.373 |
| MUDANPI | Mairin | 1376 | CPT2 | 26.373 |
| MUDANPI | Mairin | 3269 | HRH1 | 26.373 |
| MUDANPI | Mairin | 3757 | KCNH2 | 26.373 |
| MUDANPI | Mairin | 3040 | HBA2 | 26.373 |
| MUDANPI | Mairin | 476 | ATP1A1 | 26.373 |
| MUDANPI | Mairin | 3783 | KCNN4 | 26.373 |
| MUDANPI | Mairin | 1814 | DRD3 | 26.373 |
| MUDANPI | Mairin | 1374 | CPT1A | 26.373 |
| MUDANPI | Mairin | 3039 | HBA1 | 26.373 |
| MUDANPI | Mairin | 786 | CACNG1 | 26.373 |
| MUDANPI | Mairin | 125 | ADH1B | 23 |
| MUDANPI | Mairin | 1645 | AKR1C1 | 23 |
| MUDANPI | Mairin | 3156 | HMGCR | 23 |
| MUDANPI | quercetin | 506 | ATP5B | known target in DrugBank |
| MUDANPI | quercetin | 9262 | STK17B | known target in DrugBank |
| MUDANPI | quercetin | 509 | ATP5C1 | known target in DrugBank |
| MUDANPI | quercetin | 26275 | HIBCH | known target in DrugBank |
| MUDANPI | quercetin | 5294 | PIK3CG | known target in DrugBank |
| MUDANPI | quercetin | 133688 | UGT3A1 | known target in DrugBank |
| MUDANPI | quercetin | 5292 | PIM1 | known target in DrugBank |
| MUDANPI | quercetin | 498 | ATP5A1 | known target in DrugBank |
| MUDANPI | quercetin | 3055 | HCK | known target in DrugBank |
| MUDANPI | quercetin | 3356 | HTR2A | 26.373 |
| MUDANPI | quercetin | 3357 | HTR2B | 26.373 |
| MUDANPI | quercetin | 150 | ADRA2A | 26.373 |
| MUDANPI | quercetin | 3351 | HTR1B | 26.373 |
| MUDANPI | quercetin | 3358 | HTR2C | 26.373 |
| MUDANPI | quercetin | 3352 | HTR1D | 26.373 |
| MUDANPI | quercetin | 1813 | DRD2 | 26.373 |
| MUDANPI | quercetin | 1815 | DRD4 | 26.373 |
| MUDANPI | quercetin | 3350 | HTR1A | 26.373 |
| MUDANPI | quercetin | 152 | ADRA2C | 26.373 |
| MUDANPI | quercetin | 50632 | CALY | 26.373 |
| MUDANPI | quercetin | 1814 | DRD3 | 26.373 |
| MUDANPI | quercetin | 1816 | DRD5 | 26.373 |
| MUDANPI | quercetin | 1812 | DRD1 | 26.373 |
| MUDANPI | quercetin | 151 | ADRA2B | 26.373 |
| MUDANPI | quercetin | 782 | CACNB1 | 25.857 |
| MUDANPI | quercetin | 2893 | GRIA4 | 25.857 |
| MUDANPI | quercetin | 2555 | GABRA2 | 25.857 |
| MUDANPI | quercetin | 5627 | PROS1 | 25.857 |
| MUDANPI | quercetin | 8858 | PROZ | 25.857 |
| MUDANPI | quercetin | 10369 | CACNG2 | 25.857 |
| MUDANPI | quercetin | 2563 | GABRD | 25.857 |
| MUDANPI | quercetin | 1137 | CHRNA4 | 25.857 |
| MUDANPI | quercetin | 1142 | CHRNB3 | 25.857 |
| MUDANPI | quercetin | 2560 | GABRB1 | 25.857 |
| MUDANPI | quercetin | 2890 | GRIA1 | 25.857 |
| MUDANPI | quercetin | 4835 | NQO2 | 25.857 |
| MUDANPI | quercetin | 2742 | GLRA2 | 25.857 |
| MUDANPI | quercetin | 3760 | KCNJ3 | 25.857 |
| MUDANPI | quercetin | 2567 | GABRG3 | 25.857 |
| MUDANPI | quercetin | 7412 | VCAM1 | 25.857 |
| MUDANPI | quercetin | 1139 | CHRNA7 | 25.857 |
| MUDANPI | quercetin | 3763 | KCNJ6 | 25.857 |
| MUDANPI | quercetin | 2564 | GABRE | 25.857 |
| MUDANPI | quercetin | 2556 | GABRA3 | 25.857 |
| MUDANPI | quercetin | 2155 | F7 | 25.857 |
| MUDANPI | quercetin | 2030 | SLC29A1 | 25.857 |
| MUDANPI | quercetin | 779 | CACNA1S | 25.857 |
| MUDANPI | quercetin | 2565 | GABRG1 | 25.857 |
| MUDANPI | quercetin | 2158 | F9 | 25.857 |
| MUDANPI | quercetin | 2677 | GGCX | 25.857 |
| MUDANPI | quercetin | 2568 | GABRP | 25.857 |
| MUDANPI | quercetin | 57053 | CHRNA10 | 25.857 |
| MUDANPI | quercetin | 2891 | GRIA2 | 25.857 |
| MUDANPI | quercetin | 2557 | GABRA4 | 25.857 |
| MUDANPI | quercetin | 2159 | F10 | 25.857 |
| MUDANPI | quercetin | 79001 | VKORC1 | 25.857 |
| MUDANPI | quercetin | 5624 | PROC | 25.857 |
| MUDANPI | quercetin | 1136 | CHRNA3 | 25.857 |
| MUDANPI | quercetin | 2561 | GABRB2 | 25.857 |
| MUDANPI | quercetin | 775 | CACNA1C | 25.857 |
| MUDANPI | quercetin | 1141 | CHRNB2 | 25.857 |
| MUDANPI | quercetin | 776 | CACNA1D | 25.857 |
| MUDANPI | quercetin | 200909 | HTR3D | 25.857 |
| MUDANPI | quercetin | 55879 | GABRQ | 25.857 |
| MUDANPI | quercetin | 632 | BGLAP | 25.857 |
| MUDANPI | quercetin | 1135 | CHRNA2 | 25.857 |
| MUDANPI | quercetin | 8973 | CHRNA6 | 25.857 |
| MUDANPI | quercetin | 2562 | GABRB3 | 25.857 |
| MUDANPI | quercetin | 3762 | KCNJ5 | 25.857 |
| MUDANPI | quercetin | 116443 | GRIN3A | 25.857 |
| MUDANPI | quercetin | 9177 | HTR3B | 25.857 |
| MUDANPI | quercetin | 2892 | GRIA3 | 25.857 |
| MUDANPI | quercetin | 2558 | GABRA5 | 25.857 |
| MUDANPI | quercetin | 3177 | SLC29A2 | 25.857 |
| MUDANPI | quercetin | 285242 | HTR3E | 25.857 |
| MUDANPI | quercetin | 2559 | GABRA6 | 25.857 |
| MUDANPI | quercetin | 3359 | HTR3A | 25.857 |
| MUDANPI | quercetin | 2554 | GABRA1 | 25.857 |
| MUDANPI | quercetin | 1728 | NQO1 | 25.857 |
| MUDANPI | quercetin | 55584 | CHRNA9 | 25.857 |
| MUDANPI | quercetin | 89832 | CHRFAM7A | 25.857 |
| MUDANPI | quercetin | 1138 | CHRNA5 | 25.857 |
| MUDANPI | quercetin | 1143 | CHRNB4 | 25.857 |
| MUDANPI | quercetin | 3765 | KCNJ9 | 25.857 |
| MUDANPI | quercetin | 170572 | HTR3C | 25.857 |
| MUDANPI | quercetin | 2741 | GLRA1 | 25.857 |
| MUDANPI | quercetin | 154807 | VKORC1L1 | 25.857 |
| MUDANPI | quercetin | 2147 | F2 | 25.857 |
| MUDANPI | quercetin | 786 | CACNG1 | 25.857 |
| MUDANPI | sitosterol | 6646 | SOAT1 | 140.5 |
| MUDANPI | sitosterol | 290 | ANPEP | 140.5 |
| MUDANPI | sitosterol | 29881 | NPC1L1 | 140.5 |
| MUDANPI | sitosterol | 2099 | ESR1 | 122.778 |
| MUDANPI | sitosterol | 5241 | PGR | 122.778 |
| MUDANPI | sitosterol | 9971 | NR1H4 | 114.143 |
| MUDANPI | sitosterol | 7421 | VDR | 80.882 |
| MUDANPI | sitosterol | 1594 | CYP27B1 | 80.882 |
| MUDANPI | sitosterol | 2638 | GC | 55.444 |
| MUDANPI | sitosterol | 22938 | SNW1 | 55.444 |
| MUDANPI | sitosterol | 367 | AR | 48 |
| MUDANPI | sitosterol | 2908 | NR3C1 | 48 |
| MUDANPI | sitosterol | 1244 | ABCC2 | 25.857 |
| MUDANPI | sitosterol | 207 | AKT1 | 25.857 |
| MUDANPI | sitosterol | 124 | ADH1A | 25.857 |
| MUDANPI | sitosterol | 90 | ACVR1 | 25.857 |
| MUDANPI | sitosterol | 439 | ASNA1 | 25.857 |
| MUDANPI | sitosterol | 157 | ADRBK2 | 25.857 |
| MUDANPI | sitosterol | 2104 | ESRRG | 25.857 |
| MUDANPI | sitosterol | 1345 | COX6C | 25.857 |
| MUDANPI | sitosterol | 55902 | ACSS2 | 25.857 |
| MUDANPI | sitosterol | 1329 | COX5B | 25.857 |
| MUDANPI | sitosterol | 1350 | COX7C | 25.857 |
| MUDANPI | sitosterol | 4512 | COX1 | 25.857 |
| MUDANPI | sitosterol | 94 | ACVRL1 | 25.857 |
| MUDANPI | sitosterol | 107 | ADCY1 | 25.857 |
| MUDANPI | sitosterol | 22978 | NT5C2 | 25.857 |
| MUDANPI | sitosterol | 269 | AMHR2 | 25.857 |
| MUDANPI | sitosterol | 10188 | TNK2 | 25.857 |
| MUDANPI | sitosterol | 440 | ASNS | 25.857 |
| MUDANPI | sitosterol | 27 | ABL2 | 25.857 |
| MUDANPI | sitosterol | 125 | ADH1B | 25.857 |
| MUDANPI | sitosterol | 156 | ADRBK1 | 25.857 |
| MUDANPI | sitosterol | 9377 | COX5A | 25.857 |
| MUDANPI | sitosterol | 4514 | COX3 | 25.857 |
| MUDANPI | sitosterol | 1346 | COX7A1 | 25.857 |
| MUDANPI | sitosterol | 2235 | FECH | 25.857 |
| MUDANPI | sitosterol | 1327 | COX4I1 | 25.857 |
| MUDANPI | sitosterol | 5319 | PLA2G1B | 25.857 |
| MUDANPI | sitosterol | 1339 | COX6A2 | 25.857 |
| MUDANPI | sitosterol | 292 | SLC25A5 | 25.857 |
| MUDANPI | sitosterol | 3156 | HMGCR | 25.857 |
| MUDANPI | sitosterol | 5562 | PRKAA1 | 25.857 |
| MUDANPI | sitosterol | 10060 | ABCC9 | 25.857 |
| MUDANPI | sitosterol | 126 | ADH1C | 25.857 |
| MUDANPI | sitosterol | 84532 | ACSS1 | 25.857 |
| MUDANPI | sitosterol | 25 | ABL1 | 25.857 |
| MUDANPI | sitosterol | 1340 | COX6B1 | 25.857 |
| MUDANPI | sitosterol | 2172 | FABP6 | 25.857 |
| MUDANPI | sitosterol | 10939 | AFG3L2 | 25.857 |
| MUDANPI | sitosterol | 1066 | CES1 | 25.857 |
| MUDANPI | sitosterol | 65061 | CDK15 | 25.857 |
| MUDANPI | sitosterol | 445 | ASS1 | 25.857 |
| MUDANPI | sitosterol | 91 | ACVR1B | 25.857 |
| MUDANPI | sitosterol | 2180 | ACSL1 | 25.857 |
| MUDANPI | sitosterol | 19 | ABCA1 | 25.857 |
| MUDANPI | sitosterol | 293 | SLC25A6 | 25.857 |
| MUDANPI | sitosterol | 1349 | COX7B | 25.857 |
| MUDANPI | sitosterol | 4513 | COX2 | 25.857 |
| MUDANPI | sitosterol | 8883 | NAE1 | 25.857 |
| MUDANPI | sitosterol | 3683 | ITGAL | 25.857 |
| MUDANPI | sitosterol | 317 | APAF1 | 25.857 |
| MUDANPI | sitosterol | 6833 | ABCC8 | 25.857 |
| MUDANPI | sitosterol | 3066 | HDAC2 | 25.857 |
| MUDANPI | sitosterol | 238 | ALK | 25.857 |
| MUDANPI | sitosterol | 1351 | COX8A | 25.857 |
| MUDANPI | sitosterol | 8647 | ABCB11 | 25.857 |
| MUDANPI | sitosterol | 5243 | ABCB1 | 25.857 |
| MUDANPI | sitosterol | 9619 | ABCG1 | 25.857 |
| MUDANPI | sitosterol | 369 | ARAF | 25.857 |
| MUDANPI | sitosterol | 291 | SLC25A4 | 25.857 |
| MUDANPI | sitosterol | 4790 | NFKB1 | 22.373 |
| MUDANPI | sitosterol | 8644 | AKR1C3 | 22.373 |
| MUDANPI | sitosterol | 1591 | CYP24A1 | 22.373 |
| MUDANPI | sitosterol | 151306 | GPBAR1 | 22.373 |
| MUDANPI | sitosterol | 6591 | SNAI2 | 22.373 |
| MUDANPI | sitosterol | 5469 | MED1 | 22.373 |
| MUDANPI | sitosterol | 6615 | SNAI1 | 22.373 |
| MUDANPI | sitosterol | 1576 | CYP3A4 | 22.373 |
| MUDANPI | sitosterol | 793 | CALB1 | 22.373 |
| MUDANPI | sitosterol | 8074 | FGF23 | 22.373 |
| MUDANPI | sitosterol | 2672 | GFI1 | 22.373 |
| MUDANPI | sitosterol | 55915 | LANCL2 | 22.373 |
| MUDANPI | sitosterol | 54361 | WNT4 | 22.373 |
| MUDANPI | sitosterol | 6929 | TCF3 | 22.373 |
| MUDANPI | sitosterol | 581 | BAX | 22.373 |
| MUDANPI | sitosterol | 9365 | KL | 22.373 |
| MUDANPI | sitosterol | 120227 | CYP2R1 | 22.373 |
| MUDANPI | sitosterol | 5371 | PML | 22.373 |
| MUDANPI | sitosterol | 2683 | B4GALT1 | 22.373 |
| MUDANPI | sitosterol | 795 | S100G | 22.373 |
| MUDANPI | sitosterol | 1593 | CYP27A1 | 22.373 |
| MUDANPI | sitosterol | 25959 | KANK2 | 22.373 |
| MUDANPI | sitosterol | 10265 | IRX5 | 22.373 |
| MUDANPI | sitosterol | 6256 | RXRA | 22.373 |
| MUDANPI | sitosterol | 8805 | TRIM24 | 22.373 |
| MUDANPI | paeonidanin | 1576 | CYP3A4 | 48 |
| MUDANPI | paeonidanin | 3757 | KCNH2 | 48 |
| MUDANPI | paeonidanin | 28234 | SLCO1B3 | 48 |
| MUDANPI | paeonidanin | 5243 | ABCB1 | 48 |
| MUDANPI | paeonidanin | 10599 | SLCO1B1 | 48 |
| MUDANPI | kaempferol | 133688 | UGT3A1 | known target in DrugBank |
| MUDANPI | kaempferol | 3292 | HSD17B1 | 26.373 |
| MUDANPI | kaempferol | 1429 | CRYZ | 23 |
| MUDANPI | kaempferol | 79001 | VKORC1 | 23 |
| MUDANPI | kaempferol | 1728 | NQO1 | 23 |
| MUDANPI | (+)-catechin | 266629 | SEC14L3 | 48 |
| MUDANPI | (+)-catechin | 6646 | SOAT1 | 48 |
| MUDANPI | (+)-catechin | 4547 | MTTP | 48 |
| MUDANPI | (+)-catechin | 1269 | CNR2 | 48 |
| MUDANPI | (+)-catechin | 5515 | PPP2CA | 48 |
| MUDANPI | (+)-catechin | 5578 | PRKCA | 48 |
| MUDANPI | (+)-catechin | 8856 | NR1I2 | 48 |
| MUDANPI | (+)-catechin | 1268 | CNR1 | 48 |
| MUDANPI | (+)-catechin | 240 | ALOX5 | 48 |
| MUDANPI | (+)-catechin | 5516 | PPP2CB | 48 |
| MUDANPI | (+)-catechin | 23541 | SEC14L2 | 48 |
| MUDANPI | (+)-catechin | 1606 | DGKA | 48 |
| MUDANPI | (+)-catechin | 5579 | PRKCB | 48 |
| MUDANPI | (+)-catechin | 8435 | SOAT2 | 48 |
| MUDANPI | (+)-catechin | 284904 | SEC14L4 | 48 |
| SHANZHUYU | Tetrahydroalstonine | 3764 | KCNJ8 | 48 |
| SHANZHUYU | Tetrahydroalstonine | 3768 | KCNJ12 | 48 |
| SHANZHUYU | Tetrahydroalstonine | 3356 | HTR2A | 48 |
| SHANZHUYU | Tetrahydroalstonine | 3357 | HTR2B | 48 |
| SHANZHUYU | Tetrahydroalstonine | 150 | ADRA2A | 48 |
| SHANZHUYU | Tetrahydroalstonine | 3351 | HTR1B | 48 |
| SHANZHUYU | Tetrahydroalstonine | 3767 | KCNJ11 | 48 |
| SHANZHUYU | Tetrahydroalstonine | 3772 | KCNJ15 | 48 |
| SHANZHUYU | Tetrahydroalstonine | 6570 | SLC18A1 | 48 |
| SHANZHUYU | Tetrahydroalstonine | 3358 | HTR2C | 48 |
| SHANZHUYU | Tetrahydroalstonine | 3352 | HTR1D | 48 |
| SHANZHUYU | Tetrahydroalstonine | 1813 | DRD2 | 48 |
| SHANZHUYU | Tetrahydroalstonine | 6571 | SLC18A2 | 48 |
| SHANZHUYU | Tetrahydroalstonine | 1636 | ACE | 48 |
| SHANZHUYU | Tetrahydroalstonine | 3766 | KCNJ10 | 48 |
| SHANZHUYU | Tetrahydroalstonine | 3350 | HTR1A | 48 |
| SHANZHUYU | Tetrahydroalstonine | 152 | ADRA2C | 48 |
| SHANZHUYU | Tetrahydroalstonine | 1814 | DRD3 | 48 |
| SHANZHUYU | Tetrahydroalstonine | 3758 | KCNJ1 | 48 |
| SHANZHUYU | Tetrahydroalstonine | 151 | ADRA2B | 48 |
| SHANZHUYU | Tetrahydroalstonine | 3770 | KCNJ14 | 48 |
| SHANZHUYU | Tetrahydroalstonine | 495 | ATP4A | 26.373 |
| SHANZHUYU | Tetrahydroalstonine | 1723 | DHODH | 26.373 |
| SHANZHUYU | Tetrahydroalstonine | 2185 | PTK2B | 26.373 |
| SHANZHUYU | Tetrahydroalstonine | 196 | AHR | 26.373 |
| SHANZHUYU | Tetrahydroalstonine | 645 | BLVRB | 26.373 |
| SHANZHUYU | Tetrahydroalstonine | 1815 | DRD4 | 26.373 |
| SHANZHUYU | Tetrahydroalstonine | 50632 | CALY | 26.373 |
| SHANZHUYU | Tetrahydroalstonine | 1816 | DRD5 | 26.373 |
| SHANZHUYU | Tetrahydroalstonine | 55312 | RFK | 26.373 |
| SHANZHUYU | Tetrahydroalstonine | 1812 | DRD1 | 26.373 |
| SHANZHUYU | 20-Hexadecanoylingenol | 5578 | PRKCA | 122.778 |
| SHANZHUYU | 20-Hexadecanoylingenol | 5580 | PRKCD | 122.778 |
| SHANZHUYU | 20-Hexadecanoylingenol | 5734 | PTGER4 | 23 |
| SHANZHUYU | 20-Hexadecanoylingenol | 5732 | PTGER2 | 23 |
| SHANZHUYU | 20-Hexadecanoylingenol | 5733 | PTGER3 | 23 |
| SHANZHUYU | 20-Hexadecanoylingenol | 11314 | CD300A | 22.373 |
| SHANZHUYU | 20-Hexadecanoylingenol | 1113 | CHGA | 22.373 |
| SHANZHUYU | 20-Hexadecanoylingenol | 344 | APOC2 | 22.373 |
| SHANZHUYU | 20-Hexadecanoylingenol | 9928 | KIF14 | 22.373 |
| SHANZHUYU | 20-Hexadecanoylingenol | 5579 | PRKCB | 22.373 |
| SHANZHUYU | 3,4-Dehydrolycopen-16-Al | 43 | ACHE | 80.882 |
| SHANZHUYU | 3,4-Dehydrolycopen-16-Al | 590 | BCHE | 80.882 |
| SHANZHUYU | 3,4-Dehydrolycopen-16-Al | 8292 | COLQ | 55.444 |
| SHANZHUYU | 3,4-Dehydrolycopen-16-Al | 60482 | SLC5A7 | 22.373 |
| SHANZHUYU | 3,4-Dehydrolycopen-16-Al | 3084 | NRG1 | 22.373 |
| SHANZHUYU | 3,4-Dehydrolycopen-16-Al | 29958 | DMGDH | 22.373 |
| SHANZHUYU | 3,4-Dehydrolycopen-16-Al | 6496 | SIX3 | 22.373 |
| SHANZHUYU | 3,4-Dehydrolycopen-16-Al | 1401 | CRP | 22.373 |
| SHANZHUYU | 3,4-Dehydrolycopen-16-Al | 8492 | PRSS12 | 22.373 |
| SHANZHUYU | 3,4-Dehydrolycopen-16-Al | 80736 | SLC44A4 | 22.373 |
| SHANZHUYU | 3,4-Dehydrolycopen-16-Al | 501 | ALDH7A1 | 22.373 |
| SHANZHUYU | 3,4-Dehydrolycopen-16-Al | 26052 | DNM3 | 22.373 |
| SHANZHUYU | 3,4-Dehydrolycopen-16-Al | 429 | ASCL1 | 22.373 |
| SHANZHUYU | 3,4-Dehydrolycopen-16-Al | 133121 | ENPP6 | 22.373 |
| SHANZHUYU | 3,4-Dehydrolycopen-16-Al | 1006 | CDH8 | 22.373 |
| SHANZHUYU | 3,4-Dehydrolycopen-16-Al | 2339 | FNTA | 22.373 |
| SHANZHUYU | 3,4-Dehydrolycopen-16-Al | 375790 | AGRN | 22.373 |
| SHANZHUYU | 3,4-Dehydrolycopen-16-Al | 1119 | CHKA | 22.373 |
| SHANZHUYU | 3,4-Dehydrolycopen-16-Al | 55349 | CHDH | 22.373 |
| SHANZHUYU | 3,4-Dehydrolycopen-16-Al | 2902 | GRIN1 | 22.373 |
| SHANZHUYU | Aristolone | 1586 | CYP17A1 | 122.778 |
| SHANZHUYU | Aristolone | 2099 | ESR1 | 122.778 |
| SHANZHUYU | Aristolone | 5241 | PGR | 122.778 |
| SHANZHUYU | Aristolone | 4986 | OPRK1 | 122.778 |
| SHANZHUYU | Aristolone | 4306 | NR3C2 | 122.778 |
| SHANZHUYU | Aristolone | 27115 | PDE7B | 80.882 |
| SHANZHUYU | Aristolone | 8654 | PDE5A | 80.882 |
| SHANZHUYU | Aristolone | 5152 | PDE9A | 80.882 |
| SHANZHUYU | Aristolone | 5141 | PDE4A | 80.882 |
| SHANZHUYU | Aristolone | 6261 | RYR1 | 80.882 |
| SHANZHUYU | Aristolone | 5139 | PDE3A | 80.882 |
| SHANZHUYU | Aristolone | 135 | ADORA2A | 80.882 |
| SHANZHUYU | Aristolone | 3708 | ITPR1 | 80.882 |
| SHANZHUYU | Aristolone | 5145 | PDE6A | 80.882 |
| SHANZHUYU | Aristolone | 5591 | PRKDC | 80.882 |
| SHANZHUYU | Aristolone | 5293 | PIK3CD | 80.882 |
| SHANZHUYU | Aristolone | 5140 | PDE3B | 80.882 |
| SHANZHUYU | Aristolone | 5226 | PGD | 80.882 |
| SHANZHUYU | Aristolone | 5290 | PIK3CA | 80.882 |
| SHANZHUYU | Aristolone | 136 | ADORA2B | 80.882 |
| SHANZHUYU | Aristolone | 5136 | PDE1A | 80.882 |
| SHANZHUYU | Aristolone | 5144 | PDE4D | 80.882 |
| SHANZHUYU | Aristolone | 5291 | PIK3CB | 80.882 |
| SHANZHUYU | Aristolone | 3709 | ITPR2 | 80.882 |
| SHANZHUYU | Aristolone | 5153 | PDE1B | 80.882 |
| SHANZHUYU | Aristolone | 5150 | PDE7A | 80.882 |
| SHANZHUYU | Aristolone | 3710 | ITPR3 | 80.882 |
| SHANZHUYU | Aristolone | 23649 | POLA2 | 80.882 |
| SHANZHUYU | Aristolone | 5143 | PDE4C | 80.882 |
| SHANZHUYU | Aristolone | 10846 | PDE10A | 80.882 |
| SHANZHUYU | Aristolone | 5137 | PDE1C | 80.882 |
| SHANZHUYU | Aristolone | 472 | ATM | 80.882 |
| SHANZHUYU | Aristolone | 5158 | PDE6B | 80.882 |
| SHANZHUYU | Aristolone | 5142 | PDE4B | 80.882 |
| SHANZHUYU | Aristolone | 5138 | PDE2A | 80.882 |
| SHANZHUYU | Aristolone | 134 | ADORA1 | 80.882 |
| SHANZHUYU | Aristolone | 1588 | CYP19A1 | 80.882 |
| SHANZHUYU | Aristolone | 8622 | PDE8B | 80.882 |
| SHANZHUYU | Aristolone | 4907 | NT5E | 80.882 |
| SHANZHUYU | Aristolone | 3066 | HDAC2 | 80.882 |
| SHANZHUYU | Aristolone | 5146 | PDE6C | 80.882 |
| SHANZHUYU | Aristolone | 50940 | PDE11A | 80.882 |
| SHANZHUYU | Aristolone | 5151 | PDE8A | 80.882 |
| SHANZHUYU | Aristolone | 60561 | RINT1 | 55.444 |
| SHANZHUYU | Aristolone | 8737 | RIPK1 | 55.444 |
| SHANZHUYU | Aristolone | 5295 | PIK3R1 | 55.444 |
| SHANZHUYU | Aristolone | 54361 | WNT4 | 55.444 |
| SHANZHUYU | Aristolone | 6865 | TACR2 | 55.444 |
| SHANZHUYU | Aristolone | 272 | AMPD3 | 55.444 |
| SHANZHUYU | Aristolone | 1524 | CX3CR1 | 55.444 |
| SHANZHUYU | Aristolone | 100 | ADA | 55.444 |
| SHANZHUYU | Aristolone | 9001 | HAP1 | 55.444 |
| SHANZHUYU | Aristolone | 414328 | IDNK | 55.444 |
| SHANZHUYU | Aristolone | 6715 | SRD5A1 | 48 |
| SHANZHUYU | Aristolone | 367 | AR | 48 |
| SHANZHUYU | Aristolone | 2908 | NR3C1 | 48 |
| SHANZHUYU | Aristolone | 301 | ANXA1 | 48 |
| SHANZHUYU | Aristolone | 3065 | HDAC1 | 23 |
| SHANZHUYU | Aristolone | 7022 | TFAP2C | 22.373 |
| SHANZHUYU | Aristolone | 8091 | HMGA2 | 22.373 |
| SHANZHUYU | Aristolone | 8600 | TNFSF11 | 22.373 |
| SHANZHUYU | Aristolone | 84618 | NT5C1A | 22.373 |
| SHANZHUYU | Aristolone | 89 | ACTN3 | 22.373 |
| SHANZHUYU | Aristolone | 9722 | NOS1AP | 22.373 |
| SHANZHUYU | Aristolone | 6863 | TAC1 | 22.373 |
| SHANZHUYU | Aristolone | 5245 | PHB | 22.373 |
| SHANZHUYU | Aristolone | 23411 | SIRT1 | 22.373 |
| SHANZHUYU | Aristolone | 9162 | DGKI | 22.373 |
| SHANZHUYU | Aristolone | 2280 | FKBP1A | 22.373 |
| SHANZHUYU | Aristolone | 5045 | FURIN | 22.373 |
| SHANZHUYU | Aristolone | 27324 | TOX3 | 22.373 |
| SHANZHUYU | Aristolone | 10911 | UTS2 | 22.373 |
| SHANZHUYU | Aristolone | 10273 | STUB1 | 22.373 |
| SHANZHUYU | Aristolone | 9646 | CTR9 | 22.373 |
| SHANZHUYU | Aristolone | 2539 | G6PD | 22.373 |
| SHANZHUYU | Aristolone | 2779 | GNAT1 | 22.373 |
| SHANZHUYU | Aristolone | 6495 | SIX1 | 22.373 |
| SHANZHUYU | Aristolone | 5860 | QDPR | 22.373 |
| SHANZHUYU | Aristolone | 7040 | TGFB1 | 22.373 |
| SHANZHUYU | Aristolone | 286410 | ATP11C | 22.373 |
| SHANZHUYU | Aristolone | 10657 | KHDRBS1 | 22.373 |
| SHANZHUYU | Aristolone | 5532 | PPP3CB | 22.373 |
| SHANZHUYU | Aristolone | 127833 | SYT2 | 22.373 |
| SHANZHUYU | Aristolone | 51804 | SIX4 | 22.373 |
| SHANZHUYU | Aristolone | 22933 | SIRT2 | 22.373 |
| SHANZHUYU | Aristolone | 6688 | SPI1 | 22.373 |
| SHANZHUYU | Aristolone | 9456 | HOMER1 | 22.373 |
| SHANZHUYU | Aristolone | 85358 | SHANK3 | 22.373 |
| SHANZHUYU | Aristolone | 80835 | TAS1R1 | 22.373 |
| SHANZHUYU | Aristolone | 10155 | TRIM28 | 22.373 |
| SHANZHUYU | Aristolone | 7518 | XRCC4 | 22.373 |
| SHANZHUYU | Aristolone | 5469 | MED1 | 22.373 |
| SHANZHUYU | Aristolone | 4824 | NKX3-1 | 22.373 |
| SHANZHUYU | Aristolone | 7320 | UBE2B | 22.373 |
| SHANZHUYU | Aristolone | 2100 | ESR2 | 22.373 |
| SHANZHUYU | Aristolone | 10021 | HCN4 | 22.373 |
| SHANZHUYU | Aristolone | 1906 | EDN1 | 22.373 |
| SHANZHUYU | Aristolone | 773 | CACNA1A | 22.373 |
| SHANZHUYU | Aristolone | 6776 | STAT5A | 22.373 |
| SHANZHUYU | Aristolone | 7157 | TP53 | 22.373 |
| SHANZHUYU | Aristolone | 346562 | GNAT3 | 22.373 |
| SHANZHUYU | Aristolone | 92797 | HELB | 22.373 |
| SHANZHUYU | Aristolone | 1312 | COMT | 22.373 |
| SHANZHUYU | Aristolone | 65010 | SLC26A6 | 22.373 |
| SHANZHUYU | Aristolone | 150 | ADRA2A | 22.373 |
| SHANZHUYU | Aristolone | 7531 | YWHAE | 22.373 |
| SHANZHUYU | Aristolone | 1069 | CETN2 | 22.373 |
| SHANZHUYU | Aristolone | 5422 | POLA1 | 22.373 |
| SHANZHUYU | Aristolone | 1401 | CRP | 22.373 |
| SHANZHUYU | Aristolone | 1068 | CETN1 | 22.373 |
| SHANZHUYU | Aristolone | 53343 | NUDT9 | 22.373 |
| SHANZHUYU | Aristolone | 5294 | PIK3CG | 22.373 |
| SHANZHUYU | Aristolone | 650 | BMP2 | 22.373 |
| SHANZHUYU | Aristolone | 7124 | TNF | 22.373 |
| SHANZHUYU | Aristolone | 4734 | NEDD4 | 22.373 |
| SHANZHUYU | Aristolone | 183 | AGT | 22.373 |
| SHANZHUYU | Aristolone | 2241 | FER | 22.373 |
| SHANZHUYU | Aristolone | 6469 | SHH | 22.373 |
| SHANZHUYU | Aristolone | 6317 | SERPINB3 | 22.373 |
| SHANZHUYU | Aristolone | 50943 | FOXP3 | 22.373 |
| SHANZHUYU | Aristolone | 10563 | CXCL13 | 22.373 |
| SHANZHUYU | Aristolone | 8289 | ARID1A | 22.373 |
| SHANZHUYU | Aristolone | 2255 | FGF10 | 22.373 |
| SHANZHUYU | Aristolone | 3745 | KCNB1 | 22.373 |
| SHANZHUYU | Aristolone | 51176 | LEF1 | 22.373 |
| SHANZHUYU | Aristolone | 2185 | PTK2B | 22.373 |
| SHANZHUYU | Aristolone | 3981 | LIG4 | 22.373 |
| SHANZHUYU | Aristolone | 156 | ADRBK1 | 22.373 |
| SHANZHUYU | Aristolone | 4129 | MAOB | 22.373 |
| SHANZHUYU | Aristolone | 22986 | SORCS3 | 22.373 |
| SHANZHUYU | Aristolone | 153 | ADRB1 | 22.373 |
| SHANZHUYU | Aristolone | 101 | ADAM8 | 22.373 |
| SHANZHUYU | Aristolone | 143187 | VTI1A | 22.373 |
| SHANZHUYU | Aristolone | 80736 | SLC44A4 | 22.373 |
| SHANZHUYU | Aristolone | 55803 | ADAP2 | 22.373 |
| SHANZHUYU | Aristolone | 56262 | LRRC8A | 22.373 |
| SHANZHUYU | Aristolone | 2263 | FGFR2 | 22.373 |
| SHANZHUYU | Aristolone | 6790 | AURKA | 22.373 |
| SHANZHUYU | Aristolone | 4172 | MCM3 | 22.373 |
| SHANZHUYU | Aristolone | 7415 | VCP | 22.373 |
| SHANZHUYU | Aristolone | 51738 | GHRL | 22.373 |
| SHANZHUYU | Aristolone | 64919 | BCL11B | 22.373 |
| SHANZHUYU | Aristolone | 596 | BCL2 | 22.373 |
| SHANZHUYU | Aristolone | 220972 | 8-Mar | 22.373 |
| SHANZHUYU | Aristolone | 2621 | GAS6 | 22.373 |
| SHANZHUYU | Aristolone | 727 | C5 | 22.373 |
| SHANZHUYU | Aristolone | 6720 | SREBF1 | 22.373 |
| SHANZHUYU | Aristolone | 1813 | DRD2 | 22.373 |
| SHANZHUYU | Aristolone | 5350 | PLN | 22.373 |
| SHANZHUYU | Aristolone | 26052 | DNM3 | 22.373 |
| SHANZHUYU | Aristolone | 1136 | CHRNA3 | 22.373 |
| SHANZHUYU | Aristolone | 6929 | TCF3 | 22.373 |
| SHANZHUYU | Aristolone | 6714 | SRC | 22.373 |
| SHANZHUYU | Aristolone | 9361 | LONP1 | 22.373 |
| SHANZHUYU | Aristolone | 6375 | XCL1 | 22.373 |
| SHANZHUYU | Aristolone | 3326 | HSP90AB1 | 22.373 |
| SHANZHUYU | Aristolone | 11112 | HIBADH | 22.373 |
| SHANZHUYU | Aristolone | 26228 | STAP1 | 22.373 |
| SHANZHUYU | Aristolone | 3270 | HRC | 22.373 |
| SHANZHUYU | Aristolone | 2837 | UTS2R | 22.373 |
| SHANZHUYU | Aristolone | 6532 | SLC6A4 | 22.373 |
| SHANZHUYU | Aristolone | 3156 | HMGCR | 22.373 |
| SHANZHUYU | Aristolone | 28996 | HIPK2 | 22.373 |
| SHANZHUYU | Aristolone | 5530 | PPP3CA | 22.373 |
| SHANZHUYU | Aristolone | 9693 | RAPGEF2 | 22.373 |
| SHANZHUYU | Aristolone | 80312 | TET1 | 22.373 |
| SHANZHUYU | Aristolone | 9847 | C2CD5 | 22.373 |
| SHANZHUYU | Aristolone | 1437 | CSF2 | 22.373 |
| SHANZHUYU | Aristolone | 6888 | TALDO1 | 22.373 |
| SHANZHUYU | Aristolone | 624 | BDKRB2 | 22.373 |
| SHANZHUYU | Aristolone | 3757 | KCNH2 | 22.373 |
| SHANZHUYU | Aristolone | 79148 | MMP28 | 22.373 |
| SHANZHUYU | Aristolone | 3091 | HIF1A | 22.373 |
| SHANZHUYU | Aristolone | 3815 | KIT | 22.373 |
| SHANZHUYU | Aristolone | 3630 | INS | 22.373 |
| SHANZHUYU | Aristolone | 2475 | MTOR | 22.373 |
| SHANZHUYU | Aristolone | 7480 | WNT10B | 22.373 |
| SHANZHUYU | Aristolone | 6572 | SLC18A3 | 22.373 |
| SHANZHUYU | Aristolone | 10257 | ABCC4 | 22.373 |
| SHANZHUYU | Aristolone | 664 | BNIP3 | 22.373 |
| SHANZHUYU | Aristolone | 161882 | ZFPM1 | 22.373 |
| SHANZHUYU | Aristolone | 2625 | GATA3 | 22.373 |
| SHANZHUYU | Aristolone | 51366 | UBR5 | 22.373 |
| SHANZHUYU | Aristolone | 374 | AREG | 22.373 |
| SHANZHUYU | Aristolone | 1392 | CRH | 22.373 |
| SHANZHUYU | Aristolone | 132625 | ZFP42 | 22.373 |
| SHANZHUYU | Aristolone | 146850 | PIK3R6 | 22.373 |
| SHANZHUYU | Aristolone | 5196 | PF4 | 22.373 |
| SHANZHUYU | Aristolone | 477 | ATP1A2 | 22.373 |
| SHANZHUYU | Aristolone | 554 | AVPR2 | 22.373 |
| SHANZHUYU | Aristolone | 817 | CAMK2D | 22.373 |
| SHANZHUYU | Aristolone | 147 | ADRA1B | 22.373 |
| SHANZHUYU | Aristolone | 3586 | IL10 | 22.373 |
| SHANZHUYU | Aristolone | 4747 | NEFL | 22.373 |
| SHANZHUYU | Aristolone | 90226 | UCN2 | 22.373 |
| SHANZHUYU | Aristolone | 476 | ATP1A1 | 22.373 |
| SHANZHUYU | Aristolone | 6263 | RYR3 | 22.373 |
| SHANZHUYU | Aristolone | 5534 | PPP3R1 | 22.373 |
| SHANZHUYU | Aristolone | 80763 | SPX | 22.373 |
| SHANZHUYU | Aristolone | 487 | ATP2A1 | 22.373 |
| SHANZHUYU | Aristolone | 6608 | SMO | 22.373 |
| SHANZHUYU | Aristolone | 5896 | RAG1 | 22.373 |
| SHANZHUYU | Aristolone | 51762 | RAB8B | 22.373 |
| SHANZHUYU | Aristolone | 27122 | DKK3 | 22.373 |
| SHANZHUYU | Aristolone | 10768 | AHCYL1 | 22.373 |
| SHANZHUYU | Aristolone | 358 | AQP1 | 22.373 |
| SHANZHUYU | Aristolone | 7128 | TNFAIP3 | 22.373 |
| SHANZHUYU | Aristolone | 255061 | TAC4 | 22.373 |
| SHANZHUYU | Aristolone | 359787 | DPPA3 | 22.373 |
| SHANZHUYU | Aristolone | 844 | CASQ1 | 22.373 |
| SHANZHUYU | Aristolone | 2281 | FKBP1B | 22.373 |
| SHANZHUYU | Aristolone | 998 | CDC42 | 22.373 |
| SHANZHUYU | Aristolone | 3565 | IL4 | 22.373 |
| SHANZHUYU | Aristolone | 5423 | POLB | 22.373 |
| SHANZHUYU | Aristolone | 3620 | IDO1 | 22.373 |
| SHANZHUYU | Aristolone | 493 | ATP2B4 | 22.373 |
| SHANZHUYU | Aristolone | 1499 | CTNNB1 | 22.373 |
| SHANZHUYU | Aristolone | 25796 | PGLS | 22.373 |
| SHANZHUYU | Aristolone | 9368 | SLC9A3R1 | 22.373 |
| SHANZHUYU | Aristolone | 3481 | IGF2 | 22.373 |
| SHANZHUYU | Aristolone | 89781 | HPS4 | 22.373 |
| SHANZHUYU | Aristolone | 7086 | TKT | 22.373 |
| SHANZHUYU | Aristolone | 1812 | DRD1 | 22.373 |
| SHANZHUYU | Aristolone | 11033 | ADAP1 | 22.373 |
| SHANZHUYU | Aristolone | 1896 | EDA | 22.373 |
| SHANZHUYU | Aristolone | 6262 | RYR2 | 22.373 |
| SHANZHUYU | Aristolone | 947 | CD34 | 22.373 |
| SHANZHUYU | Aristolone | 3320 | HSP90AA1 | 22.373 |
| SHANZHUYU | Aristolone | 4049 | LTA | 22.373 |
| SHANZHUYU | Aristolone | 6697 | SPR | 22.373 |
| SHANZHUYU | Aristolone | 2852 | GPER1 | 22.373 |
| SHANZHUYU | Aristolone | 863 | CBFA2T3 | 22.373 |
| SHANZHUYU | Aristolone | 255022 | CALHM1 | 22.373 |
| SHANZHUYU | Aristolone | 7474 | WNT5A | 22.373 |
| SHANZHUYU | Aristolone | 6403 | SELP | 22.373 |
| SHANZHUYU | Aristolone | 91419 | XRCC6BP1 | 22.373 |
| SHANZHUYU | Aristolone | 84875 | PARP10 | 22.373 |
| SHANZHUYU | Aristolone | 3251 | HPRT1 | 22.373 |
| SHANZHUYU | Aristolone | 120425 | AMICA1 | 22.373 |
| SHANZHUYU | Aristolone | 83756 | TAS1R3 | 22.373 |
| SHANZHUYU | Aristolone | 4842 | NOS1 | 22.373 |
| SHANZHUYU | Aristolone | 54210 | TREM1 | 22.373 |
| SHANZHUYU | Aristolone | 6886 | TAL1 | 22.373 |
| SHANZHUYU | Aristolone | 210 | ALAD | 22.373 |
| SHANZHUYU | Aristolone | 5074 | PAWR | 22.373 |
| SHANZHUYU | Aristolone | 6662 | SOX9 | 22.373 |
| SHANZHUYU | Aristolone | 653 | BMP5 | 22.373 |
| SHANZHUYU | Aristolone | 5155 | PDGFB | 22.373 |
| SHANZHUYU | Aristolone | 6253 | RTN2 | 22.373 |
| SHANZHUYU | Aristolone | 967 | CD63 | 22.373 |
| SHANZHUYU | Aristolone | 818 | CAMK2G | 22.373 |
| SHANZHUYU | Aristolone | 3920 | LAMP2 | 22.373 |
| SHANZHUYU | Aristolone | 4744 | NEFH | 22.373 |
| SHANZHUYU | Aristolone | 84656 | GLYR1 | 22.373 |
| SHANZHUYU | Aristolone | 7421 | VDR | 22.373 |
| SHANZHUYU | Aristolone | 2778 | GNAS | 22.373 |
| SHANZHUYU | Aristolone | 22916 | NCBP2 | 22.373 |
| SHANZHUYU | Aristolone | 7168 | TPM1 | 22.373 |
| SHANZHUYU | Aristolone | 23533 | PIK3R5 | 22.373 |
| SHANZHUYU | Aristolone | 5046 | PCSK6 | 22.373 |
| SHANZHUYU | Aristolone | 8805 | TRIM24 | 22.373 |
| SHANZHUYU | Aristolone | 5978 | REST | 22.373 |
| SHANZHUYU | Aristolone | 1395 | CRHR2 | 22.373 |
| SHANZHUYU | Aristolone | 610 | HCN2 | 22.373 |
| SHANZHUYU | Aristolone | 5897 | RAG2 | 22.373 |
| SHANZHUYU | Aristolone | 4799 | NFX1 | 22.373 |
| SHANZHUYU | Aristolone | 80834 | TAS1R2 | 22.373 |
| SHANZHUYU | Cornudentanone | 1588 | CYP19A1 | 48 |
| SHANZHUYU | Cornudentanone | 2099 | ESR1 | 23 |
| SHANZHUYU | Cornudentanone | 5241 | PGR | 23 |
| SHANZHUYU | Cornudentanone | 367 | AR | 23 |
| SHANZHUYU | Cornudentanone | 2908 | NR3C1 | 23 |
